# Benefits from Long-Term Treatment in Irritable Bowel Syndrome

**DOI:** 10.1155/2012/936960

**Published:** 2012-01-09

**Authors:** Stefano Evangelista

**Affiliations:** Department of Preclinical Development, Menarini Ricerche SpA, Via Sette Santi 1, 50131 Firenze, Italy

## Abstract

It is known that irritable bowel syndrome (IBS) is a chronic disease of cyclic nature characterized by recurrent symptoms. IBS patients should receive, as initial therapeutic approach a short course of treatment which, if effective, has the additional value of confirming the diagnosis. Long-term treatment should be reserved to diagnosed IBS patients with recurrent symptoms. Clinical trials with stabilized therapies and new active treatments showed an improvement of the symptoms over placebo that is often time-dependent but with high relapse rates (around 40%–50% when stopping treatment). Relapse is not always immediate after stopping treatment and the recent data from OBIS trial with otilonium bromide or with psychotherapy, showed that due to different chemico-physical characteristics of the drugs or the psychosomatic impact to the disease not all treatment gave the same relapsing rate if compared to placebo. Results of IBS clinical trials with different therapies tailored to the patient needs indicate that a cyclic treatment therapy is advisable to counteract the nature of the disease.

## 1. Natural History of IBS

**Figure 1:**
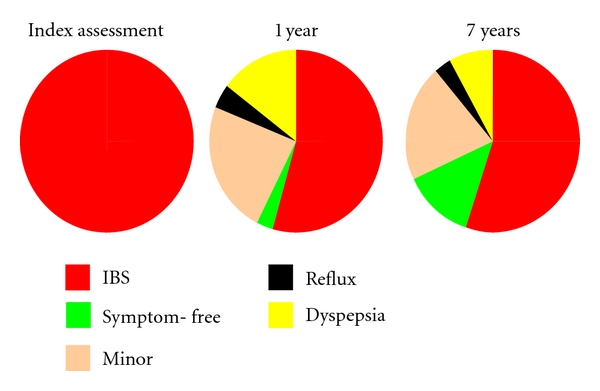
Representative diagram of the stability over time of IBS: percentage of the patients reporting IBS after 1 and 7 years from the first interview. Modified from [1].

In order to determine whether short- or long-term treatment is needed for irritable bowel syndrome (IBS), it is important to know the natural history of this disease. Various studies have looked at this. A Swedish group of researchers used a validated questionnaire to assess the course of IBS in over 1,000 patients with symptomatic IBS at the index assessment [1]. The questionnaire was administered again 1 and 7 years later, and at both time-points more than 50% of the people were still symptomatic with IBS (Figure 1). A further 25% of the patients had minor IBS symptoms, and the remaining had no longer symptoms. Similar results emerged from a survey in Olmsted County in a population of 1365 humans from which 166 were diagnosed as IBS patients [2]. In this case the follow-up was 12 years but again just over a quarter of the patients became symptom-free, whereas the remaining still had IBS symptoms. An international study followed changes in symptoms in a shorter time frame (12 weeks; [3]). Patients used an interactive telephone data entry system to report daily symptoms. The presence and duration of individual symptoms and their concomitant occurrence were determined on a total of 59 IBS patients. The main symptoms, such as pain, bloating, and change in stool form, were present in about 20% of the days. The mean duration of the symptoms was about 5 days for pain and bloating and between 1 and 2 days for the other symptoms. These results were confirmed in a large European study in which it was found that IBS patients were symptomatic for about one quarter of the days in a month [4]. Ford et al. [5] reported that of the 1402 individuals symptomatic at baseline, 404 (29%) remained in the same subgroup at 10 years while a large proportion of other patients changed subgroups altering their predominant symptoms and developing dyspepsia or gastrooesophageal reflux diseases. Symptom stability was more likely in males and older subjects. 

Indeed, there is strong evidence that the pattern of IBS symptoms is cyclical; more than half of IBS patients is still symptomatic after up to 10 years, and symptoms wax and wane within days to weeks [6].

## 2. Symptom Course during and Relapse Rate after 3 Months of Treatment 

Although IBS is clearly a chronic disease, the initial therapeutic approach is to give a short course of treatment (often 3 months), which if effective has the additional value of confirming the diagnosis. The symptom course during 3 months of treatment and the relapse rate after such treatment have been revealed by the results of several drug trials. 

A recent phase II study performed with linaclotide, an agonist of guanylate cyclase-C for the treatment of IBS patients with constipation (C), showed that all the doses utilised improved abdominal pain compared with placebo along the improvement of other intestinal habits [12]. The study was carried out on a total of 420 C-IBS and for a period of 12 weeks. The symptoms were progressively improved during the treatment time but their observation, 2 weeks after the treatment, revealed the return to baseline levels [12]. Lubiprostone, a prostaglandin E1 derivative that activates epithelial chloride channels and approved by FDA on 2008, has been tested in women with C-IBS. In one of the 3-month phase III trial (*n* = 436; [13]) the efficacy of this new drug was demonstrated over placebo group but at the conclusion of the 4-week randomised withdrawal period conducted in overall responders, 38% of patients who were randomised to continue lubiprostone and 40% of those who were randomised to placebo were reported to be monthly responders showing any difference between active treatment and placebo in this period [14].

**Figure 2:**
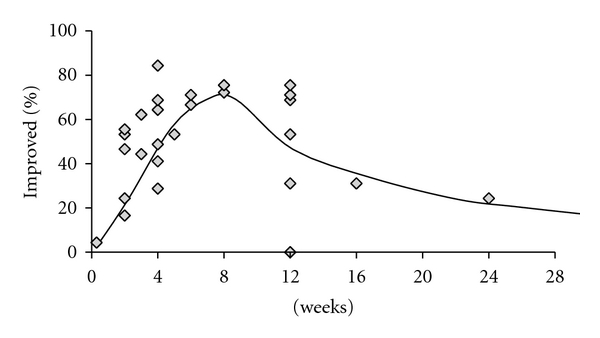
Placebo response plotted against length of trial for 27 randomised controlled trials performed during 1976–1998. There are not enough data points between 3–6 months, but it appears that the placebo response increases and then decreases with time, peaking at 8 weeks. Modified from [7].

In another IBS trial, patients were treated with the spasmolytic otilonium bromide or placebo for 4 months and the main symptoms were recorded. Not surprisingly, there were improvements in both groups, but the therapeutic gain (i.e., the difference between the improvements produced by otilonium bromide and placebo) persisted each month of treatment in terms of responder rate [15]. A similar schedule was applied for another recent study, and the results obtained on the effect of otilonium on pain frequency and bloating were found significant, and these symptoms improved progressively during the study [9]. This means that therapeutic gain is not limited to the first few weeks of treatment and it may be worthwhile continuing treatment even if it is not immediately successful. On the other hand, IBS trials are subjected to high placebo effect, typically between 30 and 60%, and this makes difficult to detect the therapeutic gain and interpretation of the results [7, 16, 17]. Two meta-analyses have shown that stringent entry criteria and an increased number of office visits are factors able to decrease the placebo response in a clinical trial [16, 17]. Being the psychosomatic part of the IBS, an important side of the disease, the reassuration and patient-practitioner relationship can give positive results. As seen in Figure 2, when the placebo response is plotted against the length of the study, a parabolic curve is drawn with the maximum of placebo response at around 6–8 weeks and a clear decline after approximately 12 weeks [7]. Therefore the placebo-controlled trials in IBS shall be realised with a time period of the active treatment superior to the 8 weeks as stated since from Rome II criteria definition [18]. 

**Figure 3:**
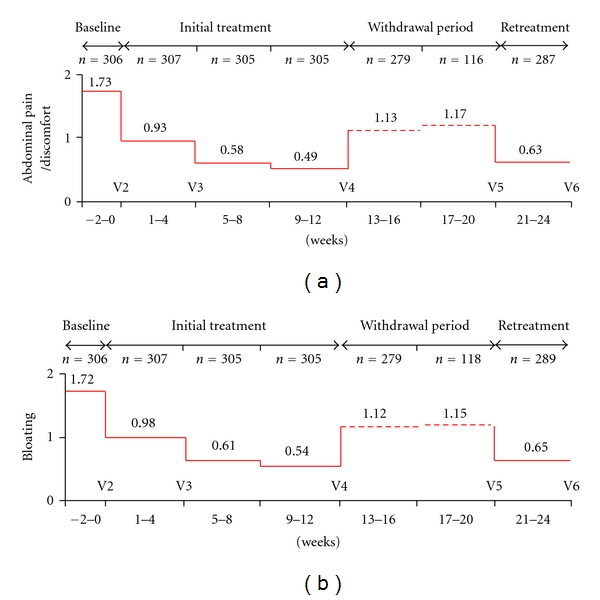
Mean abdominal pain/discomfort score (a) and bloating score (b) in patients cohort enrolled in retreatment phase with tegaserod. Modified from [8].

In another study, 623 patients were assigned to treatment with alosetron, a 5HT-3 antagonist used for diarrhoea (D) predominant IBS, or with the spasmolytic mebeverine for 3 months [19]. Symptoms recurred in both treatment groups, during the 4-week follow-up period, with the relapse rate being between 30–45%. The patients who did not relapse in this period may have relapsed later or had a spontaneous improvement, compatible with the above described natural history of IBS. The relapse rate was also recorded in a German study of patients with C-IBS treated with 5-HT4 agonist tegaserod [20]. The study was carried out on more than 300 patients, and the primary efficacy parameter was the weekly satisfactory relief of the symptoms over the past week. Patients who responded to a 3-month course of this drug were taken off the medication and followed for 1 to 2 months. If symptoms recurred, they were retreated [8]. During the treatment period symptoms improved progressively. If treatment was withdrawn, the symptoms recurred and if treatment was restarted, the symptoms improved again (Figure 3). The relapse rate in the first month of treatment withdrawal exceeded 50%. Unfortunately in this trial the placebo response was not considered, and we cannot know the real therapeutic gain obtained during the follow-up period.

**Figure 4:**
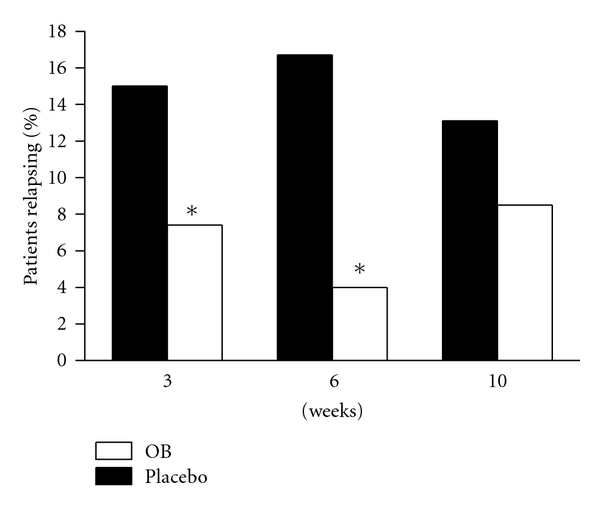
Percentage of the patients relapsing during the follow-up treatment free-period (at 3, 6, and 10 weeks) after 12 weeks treatment with placebo or otilonium bromide. *P < 0.05 as compared to respective placebo group. From OBIS trial [9].

In this regard new findings come from the above-cited OBIS study of Clavè et al. [9] with otilonium bromide. After 12 weeks of treatment, both patients treated with the active treatment (*n* = 82) or those with placebo (*n* = 80) were followed for 3, 6, or 10 weeks. Only successful treated patients were eligible for this follow-up period. The results indicate the loss of therapeutic effect of placebo and the persistence of the effect of otilonium bromide since the percentage of relapsing patients at 3 and 6 weeks was significantly higher in the placebo group (Figure 4). Probably the chemicophysical characteristics of this drug and its affinity for colonic smooth muscle [21] may be factors influencing the extension of the benefits due to the treatment that was not observed with other drugs.

In summary, the therapeutic gain from active treatment may extend beyond 4 weeks, but relapse rates are high (around 40% when stopping treatment after 3 months); relapse is not always immediate after stopping treatment.

## 3. Symptom Course and Relapse Rate after One Year of Treatment

**Figure 5:**
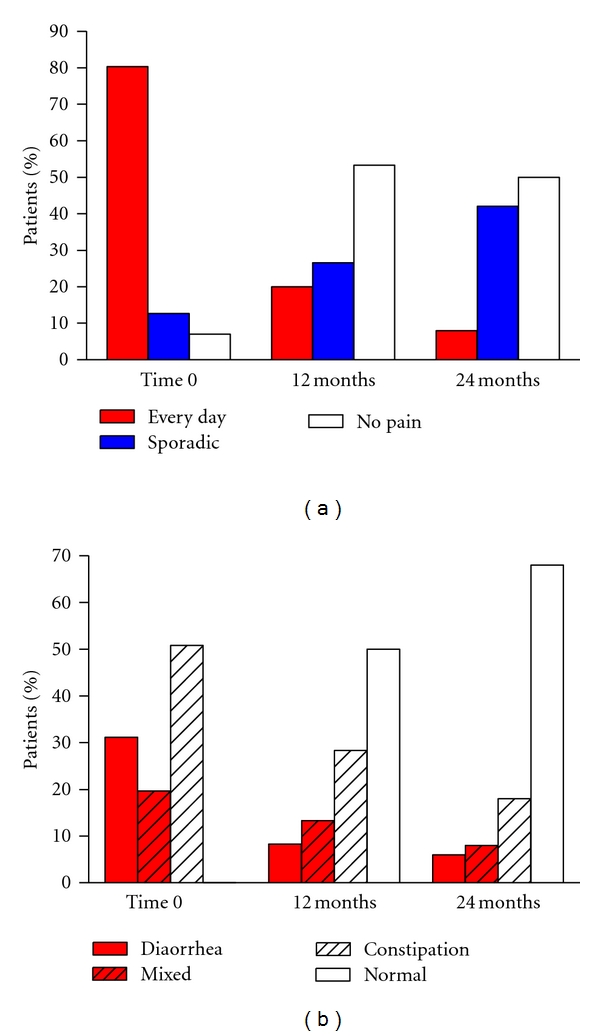
Effect of long-term treatment with otilonium bromide on pain episodes (a) and bowel habits (b) reported by patients. Modified from [10].

**Figure 6:**
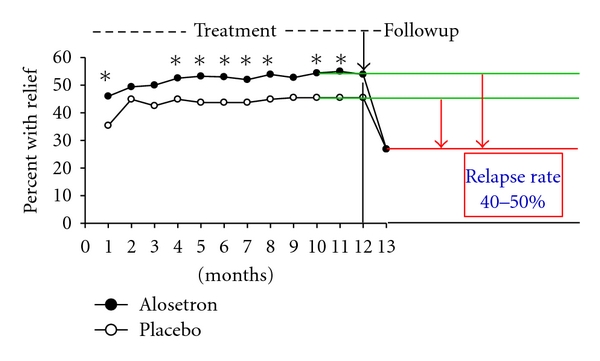
Effect of 1-year treatment with alosetron or placebo and percentage of patients with adequate relief after 1 month of followup. *P < 0.05 as compared to respective placebo group. Modified from [11].

Other studies have determined the effect of long-term treatment on the symptom course and relapse rate of IBS. In a continuation of the study with tegaserod above cited [8], the therapeutic gain was maintained over the entire 1-year period of active treatment in a total of 451 C-IBS patient who completed the trial [22]. Another study using otilonium bromide has prolonged the active treatment up to a period of 2 years [10]. In this study otilonium was compared to fiber-rich diet in 114 patients suffering from IBS. Both abdominal pain and the intestinal function improved (Figure 5) progressively during the study confirming that the long-term treatment is useful and particularly with the use of safe drugs such as otilonium that thanks to its physico-chemical characteristics cannot be absorbed by the systemic circulation and act locally in the gut like the majority of quaternary ammonium derivatives [21]. In another long-term study with the use of alosetron, 714 women with severe D-IBS were treated [11]. Randomised patients received either alosetron 1 mg (*n* = 351) or placebo (*n* = 363) twice a day during a 48-week double-blind study. The primary endpoint was the 48-week average rate of adequate relief of IBS pain and discomfort. Alosetron-treated patients had significantly greater adequate relief than placebo-treated patients (*P* < 0.05) in 9 of 12 months and significantly greater urgency control (*P* < 0.001) every month (Figure 6). Placebo effect peaked after 1 month of treatment and was stable for the other months at around 40%. It is noteworthy that when treatment was stopped, relapse occurred in nearly half of the patients after 1 month of observation. 

The benefit of active treatment can be maintained for up to 1 year or more, but the relapse rate after treatment withdrawal following long-term therapy (1-year) is still high, being around 40%.

## 4. Symptom Course during and after Psychotherapy

Interestingly, the symptom course and relapse pattern after psychotherapy seem to differ from those after drug treatment. In one trial, 101 IBS patients received standard medical therapy with or without psychotherapy administered over a 3-month period [23]. During the 3-month intervention period, the improvement was greater in the psychotherapy group than that in the control group. Subsequently, in a 1-year treatment-free followup, the improvement continued in the psychotherapy group, whereas symptoms recurred in the controls, returning to their initial state.

## 5. Approach to the Patient

The type of IBS treatment must be tailored to patients' needs. Some patients require only “single-shot” treatment. They have symptoms for 1 or 2 days and do not need an entire treatment course. Others have symptoms for a couple of days or weeks and need a course of treatment. Yet others have more or less continuous symptoms and need long-term treatment. Not all types of treatments are suitable for all applications. Spasmolytics, laxatives, and loperamide are suited for all three types of treatment: single-shot treatment, a limited course of treatment, and a long-term treatment. Prokinetics, on the other hand, are not suitable for “single-shot” treatment—they need some days to work. Antidepressants must be given for some months or even years with a look to their potential side effects. Finally, psychotherapy is administered over a couple of weeks, but it is not usually continued for months or years.

## 6. Short- and Long-Term Treatment: Advantages

There are advantages of both short-term and long-term treatment. The arguments in favour of long-term treatment are that more than half of IBS patients continue to have symptoms over many years, and the therapeutic gain of a pharmacological treatment continues for weeks or months. The relapse rate after stopping treatment is high (around 50%). Furthermore, some treatments require quite a long time to work. The arguments in favour of a short-term treatment are that about half of the patients improve over time and do not, therefore, need prolonged treatment. Although the relapse rate after treatment suspension is high, about 50% of patients do not relapse and most relapses do not occur immediately, a treatment-free interval can be gained. Some treatments, such as psychotherapy, can have long-lasting effects. The different dimensions of symptoms (intensity, frequency, and specificity) in a given patient also determine the best therapeutic approach. For example, a symptom may be of mild, moderate, or severe intensity, requiring only reassurance in some patients, an intervention in others, or in the most severe cases, multimodal intervention. Likewise, occasional, intermittent, or continuous symptoms will dictate whether on-demand treatment, a limited course of treatment or continuous treatment is necessary. The specific symptom will govern the choice of the appropriate drug. In conclusion, the drug of first choice is selected based on the dominant symptom. If this treatment is unsuccessful, the drug can be changed. If the treatment is successful, it can be suspended after a limited course. If a relapse occurs, treatment should be resumed with the same drug. If, however, the patient remains in remission he or she will not receive unnecessary treatment.
